# Qing-Re-Hua-Shi Decoction ameliorates DSS-induced colitis by modulating multiple signaling pathways and remodeling the gut microbiota and metabolite profile

**DOI:** 10.3389/fcimb.2025.1541289

**Published:** 2025-04-02

**Authors:** Wei Chen, Lin Xu, Long Wang, Yu-nan Shan, Yan Li, Jin-shui Zhu

**Affiliations:** ^1^ Department of Gastroenterology, Shanghai Sixth People’s Hospital Affiliated to Shanghai Jiao Tong University School of Medicine, Shanghai, China; ^2^ The First Clinical Medical College, Shandong University of Traditional Chinese Medicine, Jinan, China; ^3^ School of Traditional Chinese Medicine, Hubei University of Chinese Medicine, Wuhan, China

**Keywords:** Qing-Re-Hua-Shi Decoction, multi-omics analysis, ulcerative colitis, gut microbiota, metabolic homeostasis, multiple signal pathways

## Abstract

**Background:**

Clinically, Qing-Re-Hua-Shi Decoction (QRHSD) has been clinically used to treat ulcerative colitis (UC) with satisfactory outcomes and minimal side effects. However, its molecular mechanisms remain unclear.

**Purpose:**

This study investigates the effects of QRHSD on DSS-induced colitis in mice, employing multi-omics analyses, including RNA-seq transcriptomics, 16S rRNA microbiomics, non-targeted metabolomics, and network pharmacology analysis.

**Methods:**

The chemical composition of QRHSD was analyzed using quadrupole time-of-flight mass spectrometry (UPLC-Q-TOF/MS). A UC mice model was induced by 3% DSS for 7 days. The effects and mechanisms of QRHSD on UC were evaluated via hematoxylin and eosin, immunofluorescence assay, flow cytometry, western blot, RNA-seq transcriptomics, 16S rRNA microbiomics, non-targeted metabolomics, and network pharmacology. Correlation analyses and validation experiments explored links between transcriptomic, microbiome, metabolomic profiles, and UC-related clinical indices.

**Results:**

UPLC-Q-TOF/MS identified 55 compounds in QRHSD. QRHSD significantly reduced clinical activity, histological changes, and inflammatory factors in UC mice, regulated Th17/Treg balance, and enhanced intestinal barrier integrity. 16S rRNA analysis showed that QRHSD altered gut microbiota composition, increasing beneficial bacteria (e.g., *Lactobacillus*) and decreasing harmful bacteria (e.g., *Morganella*). Non-targeted metabolomics revealed 507 metabolites associated with UC amelioration, enriched in pathways like bile secretion, ABC transporters, and amino acid biosynthesis. RNA-seq analysis, network pharmacology, and experimental verification showed that QRHSD significantly regulated key signaling pathways, including PI3K/AKT, NF-κB, and MAPK signaling pathways. Finally, correlation analysis highlighted connections among UC-related clinical factors, gut microbiota, and metabolites.

**Conclusion:**

QRHSD could modulate the gut microbiota, metabolic homeostasis, and multiple signal pathways in the treatment of DSS-induced UC, revealing the mechanism of traditional Chinese medicine therapy for UC.

## 1 Introduction

Ulcerative colitis (UC) is a form of inflammatory bowel disease that affects the rectum and colon to varying degrees (Le Berre et al., 2023). Patients with UC typically experience diarrhea, abdominal pain, bloody stools, fatigue, and weight loss, significantly impairing their quality of life (Feuerstein et al., 2019; Le Berre et al., 2023). Over the past two decades, UC has become a global health challenge, with increasing incidence and prevalence rates (Le Berre et al., 2023). Currently, 5-aminosalicylic acid (5-ASA) drugs, corticosteroids, thiopurines, and biologics are the main clinical treatments for UC (Le Berre et al., 2023). Despite these therapies, 10–20% of patients still require colon surgery (Le Berre et al., 2023). Therefore, there is a need for novel and effective strategies to improve UC treatment.

The pathogenesis of UC is complex and not fully understood. Genetic susceptibility, environmental factors, epithelial barrier defects, dysregulated immune responses, and gut dysbiosis have been implicated in UC pathogenesis (Kobayashi et al., 2020; Le Berre et al., 2023). Notably, abundant evidence has highlighted the central role of the gut microbiota and metabolite disturbance in the pathogenesis of UC (Shen et al., 2018; Zhou et al., 2018; Sasaki et al., 2019; Glassner et al., 2020; Guo et al., 2020). For example, compared with healthy individuals, patients with UC typically exhibit gut microbiota imbalances, characterized by reduced gut microbiota diversity, decreased abundance of *Firmicutes*, and increased abundances of *Bacteroidetes* and *Actinobacteria* (Zakerska-Banaszak et al., 2021). Various active metabolites produced by the gut microbiota play a crucial role in maintaining intestinal barrier integrity and immune homeostasis (Hu et al., 2022). Therefore, modulating the gut microbiota and improving metabolic profile are expected to play important roles in the prevention and treatment for UC.

Herbs are promising medicines for the treatment of a wide range of diseases and have been used for centuries in China to treat UC. Evidence suggests that herbal medicine can modulate gut microbiota and metabolites, which underlies their beneficial effects (Cheng et al., 2018; Feng et al., 2019). The practice-based theory of “medicine food homology (MFH)” has existed in traditional Chinese medicine since ancient times. MFH substances provide essential nutrients to the human body while helping to prevent and treat nutritional imbalances, chronic diseases, and other health issues (Zhang et al., 2022). Qing-Re-Hua-Shi Decoction (QRHSD) is mainly composed of *Taraxacum mongolicum Hand. -Mazz.*, *Pulsatilla chinensis(Bge.)Regel*, *Sanguisorba officinalis L.*, *Areca catechu L.*, *Sophora japonica L.*, *Bletilla striata(Thunb.)Reichb.f.*, *Paeonia veitchii Lynch*, and *Euryle ferox Salisb.* Clinically, we have applied QRHSD to treat UC patient with satisfactory results and low side effects (Zhou et al., 2024). Notably, most of the herbs in QRHSD, such as *Taraxacum mongolicum Hand. -Mazz.*, *Areca catechu L.*, *Sophora japonica L.*, and *Euryle ferox Salisb.* are MFH substances with both medicinal and food uses. The main ingredients of QRHSD possess multiple biological functions, including anti-inflammatory, immunomodulatory, and gut microbiota-regulating properties. For example, our previous study demonstrated that *Taraxacum officinale* improved intestinal inflammation and regulated the gut microbiota (Chen et al., 2019). Pulsatilla Decoction (mainly composed of *Pulsatilla*) has good therapeutic effects in UC mice by modulating immune function, gut microbiota, and short-chain fatty acids derived from gut bacteria (Xuan-Qing et al., 2021; Niu et al., 2023). Despite its clinical effectiveness, the mechanisms of QRHSD remain undefined, limiting its broader application. Given that UC pathogenesis involves complex interactions between the immune system, gut microbiota, and metabolites, a multi-omics approach integrating transcriptomics, microbiomics, and metabolomics provides a comprehensive method to elucidate the therapeutic mechanisms of QRHSD.

In this study, we first analyzed the components of QRHSD using quadrupole time-of-flight mass spectrometry (UPLC-Q-TOF/MS). For the first time, we employed a multi-omics approach integrating RNA-seq transcriptomics, 16S rRNA microbiome profiling, non-targeted metabolomics, and network pharmacology to investigate the effects of QRHSD on the transcriptome, microbiome, and metabolome in a mouse model of UC. Furthermore, we explored the relationships among key intestinal flora, metabolites, and UC-related clinical parameters. These findings provide an experimental basis for the clinical application of QRHSD in UC treatment.

## 2 Materials and methods

### 2.1 Drugs and reagents

DSS was purchased from MP Biomedicals (Santa Ana, CA, United States, cat# 160110), The following antibodies were used in this study: anti-TNF-α (GB11188, Serbicebio, China), anti-IL-6 (GB11117, Serbicebio, China), anti-IL-1β (GB13463, Serbicebio, China), FITC-CD4 (Biolegend, 201,505), PE-IFN-γ (Biolegend, 507,810), APC-IL-17A (eBioscience, 17–7177-81), PE-CD25 (Biolegend, 202,105), AF647-FOXP3 (Biolegend, 320,014). NF-κB p65 (Proteintech, 10745-1-AP), Phospho-NF-κB p65 (Affinity, AF2006), AKT (Proteintech, 10176-2-AP), Phospho-AKT (Proteintech, 28731-1-AP), PI3K (Cloud-Clone Corp., PAJ829Hu01), Phospho-PI3K (Affinity, AF3241), p38MAPK (Proteintech, 51115-1-AP), Phospho-p38MAPK (Affinity, AF4001), ZO-1 (Affinity, AF5145), Occludin (Affinity, DF7504). The herbal formula QRHSD, composed of *Taraxacum mongolicum Hand. -Mazz.*30g, *Pulsatilla chinensis(Bge.)Regel* 30g, *Sanguisorba officinalis L.*20g, *Areca catechu L.*20g, *Sophora japonica L.*30g, *Bletilla striata(Thunb.)Reichb.f.*15g, *Paeonia veitchii Lynch* 20g, and *Euryle ferox Salisb* 15g, were purchased from Traditional Chinese Medicine Department of the First Affiliated Hospital of Shandong University.

### 2.2 Animal experiments

All animals were bought from Shanghai SLAC Laboratory Animal Co. Ltd. and housed in the laboratory animal center of the first hospital affiliated with Shandong First Medical University & Shandong Provincial Qianfoshan Hospital. The animals were maintained under standard environmental conditions, including a 12-hour light/dark cycle, room temperature of 25 ± 2°C, and relative humidity of 50–55%. All animal experiments were approved by the Ethical Review Committee of The First Hospital Affiliated with Shandong First Medical University& Shandong provincial Qianfoshan Hospital (application number: 2024040901). After one week of acclimatization, male C57BL/6 mice (6–8 weeks old) were randomly allocated into four groups (n = 7 per group): Control group, DSS+ water group, DSS+ QRHSD (H) group (high-dose 18g/kg) and DSS+ QRHSD (L) group (low-dose 9g/kg). The dosage of QRHSD for mice was determined based on a previously established dose conversion method (Wojcikowski and Gobe, 2014). To induce colitis, C57BL/6 mice were administered with 3.0% DSS in their drinking water for 7 days. Mice in the treatment groups received daily oral gavage of the corresponding dose of QRHSD, while mice in the Control and DSS+water groups were administered an equivalent volume of solvent. The body weight and disease activity index (DAI) scores were evaluated daily throughout the experimental period. After 7 days of treatment, mice were euthanized under isoflurane anesthesia.

### 2.3 Histological assessment

For histological analysis, distal colon tissues from mice were fixed with 4% paraformaldehyde, embedded in paraffin, and sectioned into thin slices. The sections were then stained with hematoxylin and eosin (H&E) to evaluate tissue morphology. Pathological changes in the colon tissues were evaluated under a light microscopy, and histological scores were calculated according to a previously established protocol (Li et al., 2022).

### 2.4 Immunofluorescence assay

Paraffin-embedded sections of distal colon tissues were deparaffinized and treated with 3% hydrogen peroxide for 25 minutes in the dark to quench endogenous peroxidase activity. The sections were then blocked with BSA for 30 minutes at room temperature. Subsequently, the sections were incubated with primary antibodies overnight at 4°C in the dark. After washing, the corresponding secondary antibodies were then added separately and incubated for 1 hour at room temperature. Finally, the nuclei were counterstained with DAPI for 10 minutes at room temperature. The stained sections were mounted with coverslips and visualized under a fluorescence microscope for image.

### 2.5 Alcian blue/periodic acid Schiff staining

First, paraffin sections were deparaffinized with xylene and absolute ethanol, and then stained according to the AB/PAS kit instructions: sections were stained with AB-PAS C for 15 minutes and rinsed with tap water until colorless. The sections were then stained with AB/PAS B for 15 minutes, rinsed with tap water, and rinsed twice with distilled water. Sections were stained using AB/PAS A at room temperature for 30 minutes in the dark and then rinsed for 5 minutes. After dehydration and sealing, images were observed under a microscope.

### 2.6 Western blot analysis

Tissue proteins were extracted using RIPA lysis buffer (#P0013C, Beyotime) supplemented with protease inhibitor (#ST505, Beyotime) and phosphatase inhibitor (#P1045, Beyotime). The total protein concentration was quantified using a BCA protein assay kit (#PK10026, Proteintech) according to the manufacturer’s instructions. Protein samples were separated electrophoretically on a 10% sodium dodecyl sulfate-polyacrylamide (SDS-PAGE) gel and equal amounts of total protein were loaded into each lane. Proteins were then transferred to a 0.45 μm pore size polyvinylidene difluoride (PVDF) membrane (Merck Millipore, USA) by wet transfer. The membranes were blocked with 5% skim milk powder for 1 h at room temperature and then incubated overnight at 4°C with the primary antibodies. Following several washes, the membranes were incubated with secondary antibodies for 1 h at room temperature. Finally, the protein bands were visualized using a luminescence imaging system (Tanon).

### 2.7 UPLC-Q-TOF/MS

Waters H-Class ultra-performance liquid chromatography (Waters Technology Co., Ltd.) and AB Sciex Triple TOF^®^ 4600 high-resolution mass spectrometry (SCIEX, Framingham, USA) were used to analyze the ingredients of QRHSD. Briefly, 0.5 ml of QRHSD sample was taken, three times volume diluted with 50% methanol, shaken well, centrifuged at high speed (12,000 rpm) for 5 minutes, and the supernatant was taken.

The chromatographic column was Waters ACQUITY UPLC^®^ CSH C18 (2.1×100 mm, 2.7µm) with a column temperature of 30°C, a flow rate of 0.3 ml/min, and an injection volume of 2 µl. The detection wavelength in chromatographic conditions was 190 nm -400 nm. The flow ratio is as follows: phase A acetonitrile and phase B 0.1% formic acid aqueous solution, with gradients shown in **Supplementary Table 1**. Set the mass spectrometry detection mode to ESI-Negative/Positive ion mode. The data acquisition software was Analyst TF 1.7.1, and the data processing software was Peakview 1.2. In the identification process, the mass spectrometry data was preferentially matched with the Natural Products HR-MS/MS Spectral Library 1.0 database, and the compounds were preliminarily screened based on the score information of the peaks, and were further confirmed on the basis of the primary and secondary information of the peaks. The compounds were initially screened based on the score information of each peak, and further confirmed based on the primary and secondary information of each peak.

### 2.8 Transcriptomic analysis

Total RNA was extracted from colon tissues using TRIzol reagent (Invitrogen, Carlsbad, CA, USA) according to the manufacturer’s instructions. The quality and integrity of the extracted RNA were assessed using a NanoDrop ND-1000 (NanoDrop, Wilmington, DE, USA) and Bioanalyzer 2100 (Agilent, CA, USA). After RNA extraction, purification, and library construction, we performed the 2×150bp paired-end sequencing (PE150) on an Illumina Novaseq™ 6000 (LC-Bio Technology CO., Ltd., Hangzhou, China) following the vendor’s recommended protocol. Quality control of the raw sequencing data was performed using fastp (https://github.com/OpenGene/fastp) software. Sequencing data were compared to the genome using HISAT2 (https://ccb.jhu.edu/software/hisat2). StringTie was used to perform expression level for mRNAs by calculating FPKM (FPKM = [total_exon_fragments/mapped_reads(millions) × exon_length(kB)]). The differentially expressed mRNAs were selected with fold change > 2 or fold change < 0.5 and with parametric F-test comparing nested linear models (p value < 0.05) by R package edgeR (https://bioconductor.org/packages/release/bioc/html/edgeR.html). Finally, genes were analyzed for GO and KEGG enrichment using DAVID software (https://david.ncifcrf.gov/).

### 2.9 Network pharmacology analysis

The Canonical SMILES of QRHSD components were obtained from the PubChem Database (https://pubchem.ncbi.nlm.nih.gov/). Potential molecular targets of QRHSD components were predicted using the Swiss Target Prediction Database (http://www.swisstargetprediction.ch/) and TTD (https://db.idrblab.net/ttd/).Target proteins corresponding to the compounds were standardized in UniProt (https://www.uniprot.org/). UC-associated targets were obtained from the OMIM Database (http://omim.org/), Therapeutic Targets Database (http://bidd.nus.edu.sg/group/cjttd/), Drugbank (https://go.drugbank.com/), Disgenet (https://ngdc.cncb.ac.cn/databasecommons/) and GeneCards (https://www.genecards.org/). These resources were searched using the keyword ‘ulcerative colitis’. Following the removal of redundant entries, we obtained a curated list of target genes related to UC. Then, Cytoscape 3.10.0 software (https://cytoscape.org/) was used, and the ‘QRHSD compound-target’ and ‘UC-target’ networks were constructed. The common protein targets of UC and QRHSD compounds were screened out for further analysis by the Kyoto Encyclopedia of Genes and Genomes (KEGG) and Gene Ontology (GO). In this study, KEGG pathway analysis and GO analysis were performed by linking targets to Integrated (https://metascape.org/gp/index.html#/main/step1).

### 2.10 Intestinal microbiota analysis

Total genomic DNA were extracted from fecal samples using the OMEGA Soil DNA Kit (M5635-02) (Omega Bio-Tek, Norcross, GA, USA). PCR amplification of the bacterial 16S rRNA genes V3–V4a region was performed using the forward primer ACTCCTACGGGAGGCAGCA and the reverse primer GGACTACHVGGGTWTCTAAT. PCR amplicons were purified with Vazyme VAHTSTM DNA Clean Beads (Vazyme, Nanjing, China) and quantified using the Quant-iT PicoGreen dsDNA Assay Kit (Invitrogen, Carlsbad, CA, USA). The sequencing was performed using the Illumina NovaSeq platform with NovaSeq 6000 SP Reagent Kit (500 cycles) at Shanghai Personal Biotechnology Co., Ltd (Shanghai, China). Microbiome bioinformatics were performed with QIIME2 2022.11 (Bolyen et al., 2018) with slight modification according to the official tutorials (https://docs.qiime2.org/2022.11/tutorials/). Non-singleton amplicon sequence variants (ASVs) were aligned with mafft (Katoh et al., 2002) and used to construct a phylogeny with fasttree2 (Price et al., 2009). Alpha-diversity metrics and beta-diversity metrics were estimated. Linear discriminant analysis (LDA) effect sizes were used to analyze the microbiota features among the experimental groups.

### 2.11 Untargeted metabolomics analysis

Metabolomic profiling of intestinal content was conducted using a non-targeted metabolomics approach based on liquid chromatography and mass spectrometry (LC-MS) system. Briefly, metabolites were extracted and analyzed using a Vanquish (Thermo Fisher Scientific) ultra-high performance liquid chromatography for polar metabolites. The compounds were separated by a Waters ACQUITY UPLC BEH Amide (2.1 mm × 50 mm, 1.7 μ m) liquid chromatography column. After being converted to mzXML format by Proteowizard software (v3.0.8789), the data was processed using the XCMS software package in R. The sample data were analyzed by principal component analysis (PCA), Partial Least Squares Discriminant Analysis (PLS-DA), and Orthogonal partial leastsquares discriminant (OPLS-DA) downscaling using the R software package Ropls (Thévenot et al., 2015), respectively. Statistical test analysis for multiple comparisons was performed using PMCMRplus (V1.9.6) to obtain significant p-value results. Cluster analysis of differential metabolite abundance values was performed using cluster analysis via the Pheatmap program package in R (V1.0.12) to plot heatmaps and trend analysis; Venn diagrams and Upset plots of differential substances for two or different multiple group comparisons were plotted using VennDiagram (V1.7.3) and UpSetR (V1.4.0); and corrplot was enabled (V4.0.3) for correlation analysis of differential metabolites; and functional analysis of differential metabolites, mainly through clusterProfiler (V4.6.0) for KEGG enrichment analysis of differential substances, to obtain information of significantly enriched metabolic pathways.

### 2.12 Flow cytometry analysis

Colon tissue was mechanically dissociated and passed through a 70 μm cell strainer to generate a single-cell suspension of mononuclear cells (MNCs). 100 μl of colon MNC suspension was taken and labeled with appropriate antibodies. Intracellular cytokine staining was stimulated with Cell Stimulation Cocktail (#00-4975-93, eBioscience) for 5 h, followed by staining with Zombie (#423103, Biolegend) for 30 min at 4°C, surface staining with CD4 (#100406, Biolegend) for 30 min, and then staining with anti-IL-17A (# 506916; eBioscience), anti-IFN-γ (#505808 Biolegend) for intracellular antigen and antibody staining, respectively. To stain Treg cells, cells were first stained with surface markers CD4 and CD25 (#101904; Biolegend), followed by fixation and permeabilization with Fixation and Permeabilization Solution (#554722, BD) and Perm/Wash Buffer (#554723, BD). Finally, Foxp3 Antibody (#320014, Biolegend) was added for 30 minutes in the dark. Cells were stained by a BD AriaII flow cytometer and Flowjo software (TreeStar Inc., Ashland, OR, USA).

### 2.13 Statistical analysis

All experimental data were presented as the means ± SD. Statistical analysis was performed by GraphPad Prism 7 (GraphPad Software, La Jolla, CA). For comparisons between two groups, an unpaired Student’s *t*-test was used, assuming equal variance and a two-tailed distribution. For comparisons involving three or four groups, one-way ANOVA was performed, followed by Tukey’s *post-hoc* test to adjust for multiple comparisons and control the family-wise error rate. A *P* values of*<*0.05 were considered statistically significant.

## 3 Results

### 3.1 Identification of the chemical constituents of QRHSD

**Figure 1:**
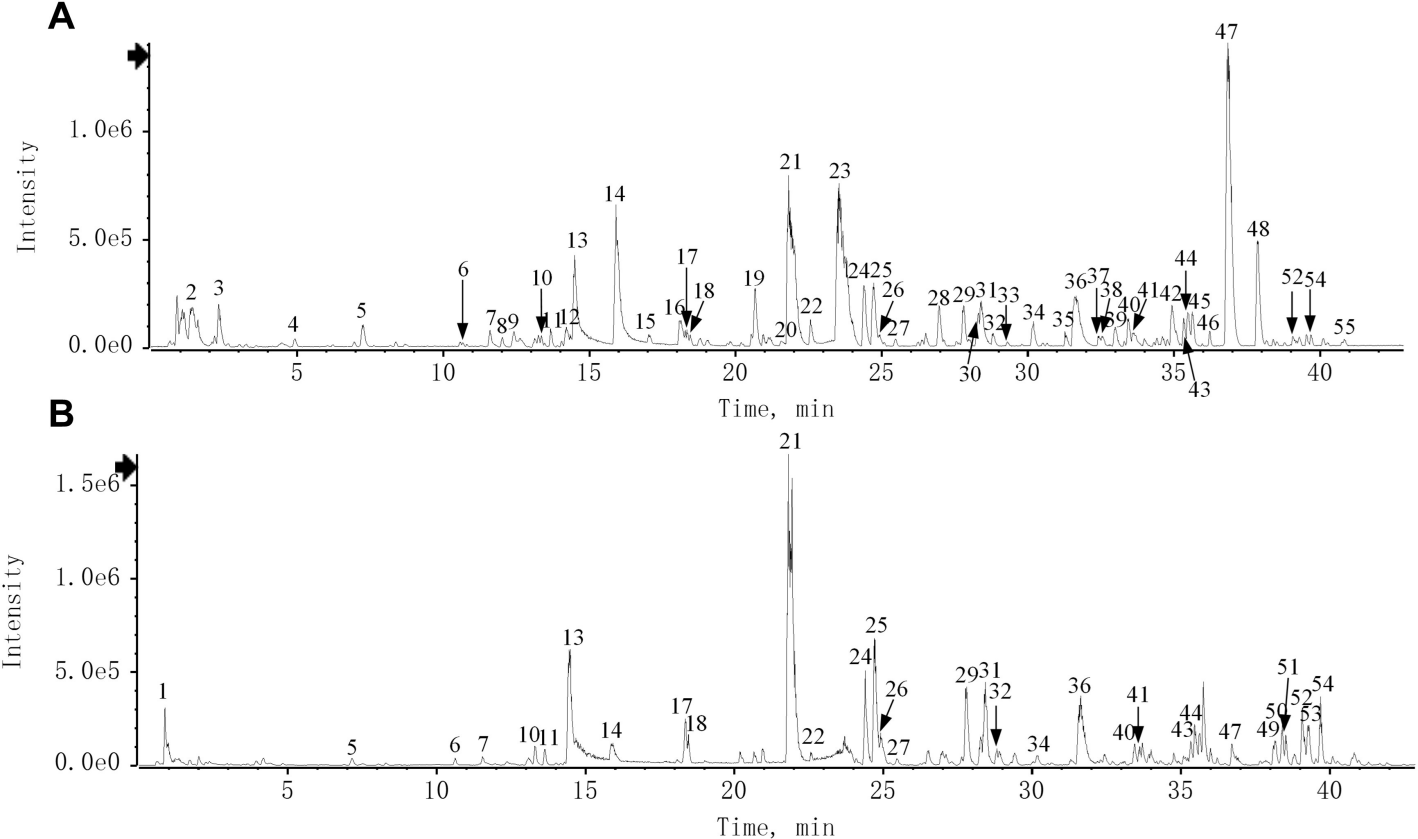
Total ion chromatogram of QRHSD in negative **(A)** and positive **(B)** ion mode of UPLC-HRMS.

The chemical characteristics of QRHSD were identified by UPLC-Q-TOF/MS, and 55 compounds were detected. The positive and negative ion mode chromatograms of QRHSD are shown in **Figure 1**, and a list of these 55 compounds is provided in **Table 1**. The identified compounds include arecoline, gallic acid, oxypagoniflorin, caffeic acid, ziyuglycoside I, albiflorin, chicoric acid, and others.

**Table 1:** Results of UPLC-Q-TOF-MS analysis of QRHSD.

Peak name	Time (min)	Adduct ion	m/z (experiment)	Compound name
1	0.87	[M+H]^+^	156.1021	Arecoline
2	1.41	[M-H]^-^	191.0209	Citric acid
3	2.32	[M-H]^-^	169.0154	Gallic acid
4	4.94	[M-H]^-^	153.0207	Protocatechuic acid
5	7.26	[M-H]^-^	345.0845	Methyl-6-O-galloyl-β-D-glucopyranoside
6	10.68	[M-H]^-^	495.1522	Oxypaeoniflorin
7	11.61	[M-H]^-^	289.0728	Catechin
8	12.02	[M-H]^-^	495.1528	Oxypaeoniflorin
9	12.43	[M-H]^-^	179.0364	Caffeic acid
10	13.31	[M+H]^+^	433.1338	Licoagroside B
11	13.64	[M+H]^+^	565.1752	Soyamaloside C
12	14.23	[M-H]^-^	353.0897	Chlorogenic acid
13	14.49	[M+FA-H]^-^	525.164	Albiflorin
14	15.92	[M+FA-H]^-^	525.1655	Paeoniflorin
15	17.04	[M-H]^-^	325.0587	Fertaric acid isomer
16	18.1	[M-H]^-^	325.0582	Fertaric acid
17	18.35	[M-H]^-^	755.2089	Quercetin-3-rutinoside-7-rhamnoside
18	18.46	[M-H]^-^	771.2039	Quercetin-3-O-rutinoside-7-O-glucoside
19	20.68	[M-H]^-^	619.2274	Dactylorhin E
20	21.57	[M-H]^-^	300.9999	Ellagic acid
21	21.83	[M-H]^-^	609.1489	Rutin
22	22.57	[M-H]^-^	463.0907	Hyperoside
23	23.56	[M-H]^-^	277.0042	Gallic acid ethyl ester sulfate
24	24.41	[M-H]^-^	593.1534	Kaempferol-3-O-rutinoside
25	24.73	[M-H]^-^	623.1656	Narcissoside
26	24.93	[M-H]^-^	609.1501	Quercetin-3-O-β-D-glucosyl-(1→2)-rhamnoside
27	25.47	[M-H]^-^	577.1593	Apigenin-7-O-rutinoside
28	26.98	[M-H]^-^	661.2399	Dactylorhin E+acetylation
29	27.82	[M-H]^-^	577.16	/
30	28.31	[M-H]^-^	473.0751	Chicoric acid
31	28.41	[M+FA-H]^-^	771.2757	Militarine
32	28.82	[M+FA-H]^-^	975.3402	Gymnoside III
33	29.32	[M-H]^-^	461.0758	3,4’-Di-O-methylellagic acid -4-β-D-xylopyranoside
34	30.19	[M+FA-H]^-^	493.2327	Geranyl-1-O-furanyl-arabinose-(1→6)-β-D-glucopyranoside
35	31.31	[M+2FA-2H]^2-^	736.3391	Patrinia saponin H3
36	31.66	[M-H]^-^	301.0366	Quercetin
37	32.43	[M+2FA-2H]^2-^	736.3389	Hederacolchiside F
38	32.56	[M-H]^-^	487.0916	Caffeoyl-feruloyltartaric acid
39	33	[M-H]^-^	394.973	3-O-methylellagic acid sulfate
40	33.44	[M+FA-H]^-^	629.1916	Benzoylpaeoniflorin
41	33.62	[M+2FA-2H]^2-^	655.3122	Anemoside B4
42	34.94	[M-H]^-^	501.108	Diferuloyltartaric acid
43	35.34	[M-H]^-^	285.0427	Kaempferol
44	35.49	[M-H]^-^	315.0528	Isorhamnetin
45	35.64	[M+FA-H]^-^	811.4554	Ziyuglycoside I
46	36.22	[M-H]^-^	287.225	Dioxy hexadecanoic acid
47	36.86	[M-H]^-^	408.9907	3,4’-Di-O-methylellagic acid sulfate
48	37.88	[M-H]^-^	423.005	3,3’,4’-O-Trimethylellagic acid Sulfate
49	38.17	[M+H]^+^	666.3146	/
50	38.41	[M+H]^+^	666.3125	/
51	38.52	[M+H]^+^	943.5281	Pulsatilloside C
52	39.09	[M+H]^+^	797.4698	Pulsatilloside B
53	39.3	[M+H]^+^	680.329	/
54	39.69	[M+H]^+^	680.329	/
55	40.84	[M+FA-H]^-^	795.4573	Anemoside A3

### 3.2 QRHSD alleviated DSS-induced experimental colitis in mice

**Figure 2:**
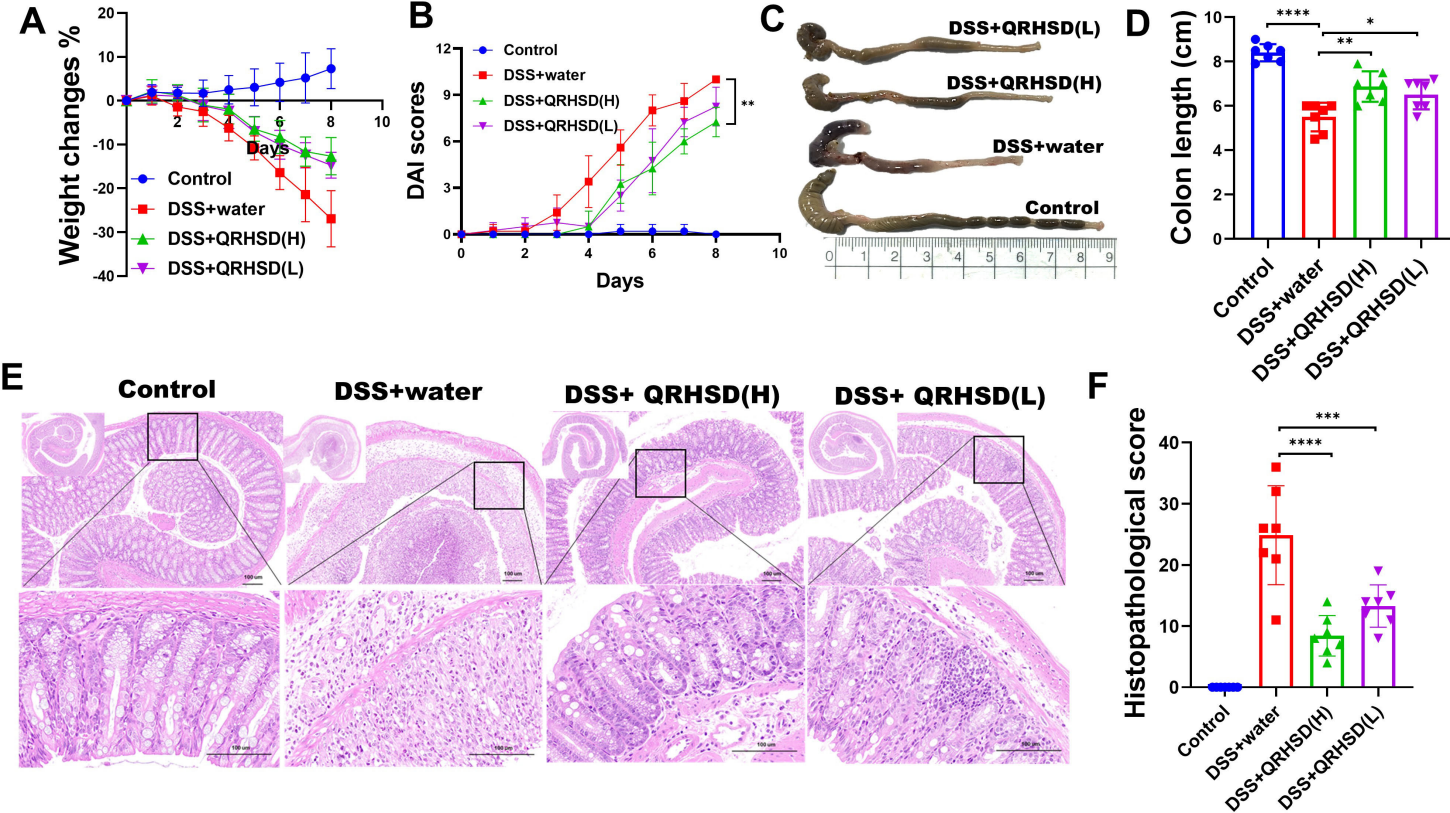
QRHSD alleviated DSS-induced experimental colitis in mice. **(A)** Bodyweight changes were measured daily. **(B)** DAI changes were compared. **(C)** The colon lengths were measured at day 7. **(D)** Representative schematic of the colon from each group of mice. **(E)** Representative HE staining of distal colon tissues of each group. **(F)** Histopathological scores of colons in each group. Data are expressed as means ± SD. n = 7 mice/group, *P < 0.05, **P < 0.01, *** *P* < 0.001, and ****P <0.0001.

To investigate the effect of QRHSD on UC, we administered high and low doses of QRHSD daily to colitis mice by oral gavage. We monitored and recorded the body weight and activity levels of the mice in each group. After 7 days of treatment, colon tissues were collected, and their lengths were measured. Histological analysis was performed using hematoxylin and eosin (HE) staining. We found that QRHSD significantly ameliorated the DSS-induced decrease in body weight (**Figure 2A**). Moreover, QRHSD treatment reduced the elevated disease activity index (DAI) score during disease progression in colitis mice (**Figure 2B**). In addition, compared with the DSS + water group, the colon shortening was significantly reduced in the QRHSD treatment groups (**Figures 2C, D**). HE staining revealed that QRHSD treatment markedly improved DSS-induced pathological changes in the colon. Specifically, the QRHSD-treated group exhibited well-structured crypts, reduced inflammation, less mucosal damage, and lower pathological scores (**Figures 2E, F**), indicating that QRHSD alleviated UC symptoms.

### 3.3 QRHSD inhibited intestinal inflammation and restored Th17/Treg balance in UC mice

**Figure 3:**
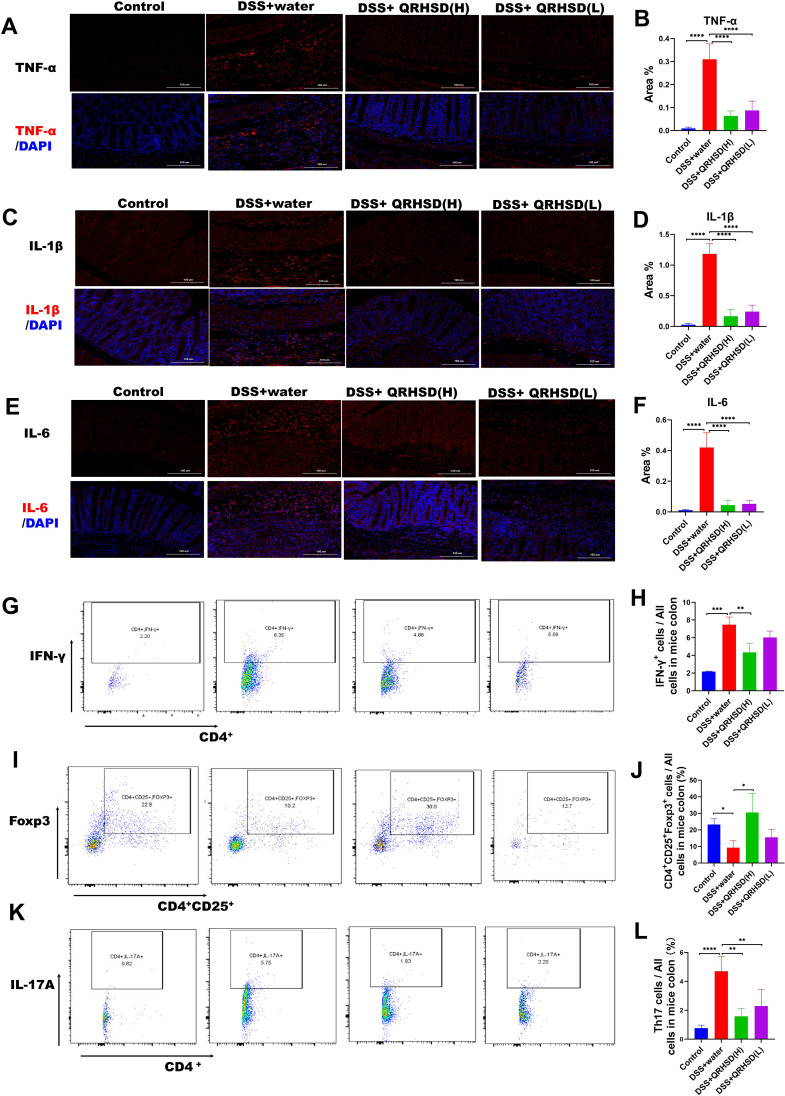
QRHSD inhibited intestinal inflammation and maintained the balance of Th17/Treg in UC mice. Representative pictures and analysis of TNF-α (**A, B**), IL-1β (**C, D**), and IL-6 (**E, F**) expression in colon tissues of each group. CD4^+^IFN-γ^+^ **(G)**, CD4^+^CD25^+^Foxp3^+^ Tregs **(I)**, and CD4^+^IL-17A^+^Th17 cells **(K)** were examined by FACS. The percentages of CD4^+^IFN-γ^+^ **(H)**, CD4^+^CD25^+^Foxp3^+^ Tregs **(J)**, and CD4^+^IL-17A^+^Th17 cells **(L)** in the four groups were analyzed. Data are expressed as means ± SD. n = 7 mice /group, * *P* < 0.05, **P < 0.01, *** *P* < 0.001, and ****P <0.0001.

Given that excessive secretion of proinflammatory cytokines is closely associated with intestinal inflammation of UC, We measured the levels of inflammatory cytokines in colonic tissues using immunofluorescence and found that the levels of TNF-α, IL-1β, and IL-6 were significantly reduced in QRHSD-treated groups (**Figures 3A–F**). Additionally, fluorescence-activated cell sorting (FASC) was used to analyze the proportion of CD4^+^IFN-γ^+^ cells in colonic tissues. As shown in **Figures 3G, H**, QRHSD downregulated the proportion of CD4^+^IFN-γ^+^cells. Th17/Treg imbalance is a key pathogenic mechanism in UC (Chang et al., 2021). Here, flow cytometry was performed to quantify CD4^+^CD25^+^Foxp3^+^ Tregs and CD4^+^IL-17A^+^Th17 cells in colonic tissues. The results showed that QRHSD treatment increased the proportion of CD4^+^CD25^+^Foxp3^+^ Tregs in UC mice model (**Figures 3I, J**) and decreased the proportion of CD4^+^IL-17A^+^Th17 cells (**Figures 3K, L**) in the UC mouse model. These results suggested that QRHSD inhibited intestinal inflammation and restored Th17/Treg cell balance in UC mice model.

### 3.4 QRHSD strengthened the intestinal barrier in UC mouse model

**Figure 4:**
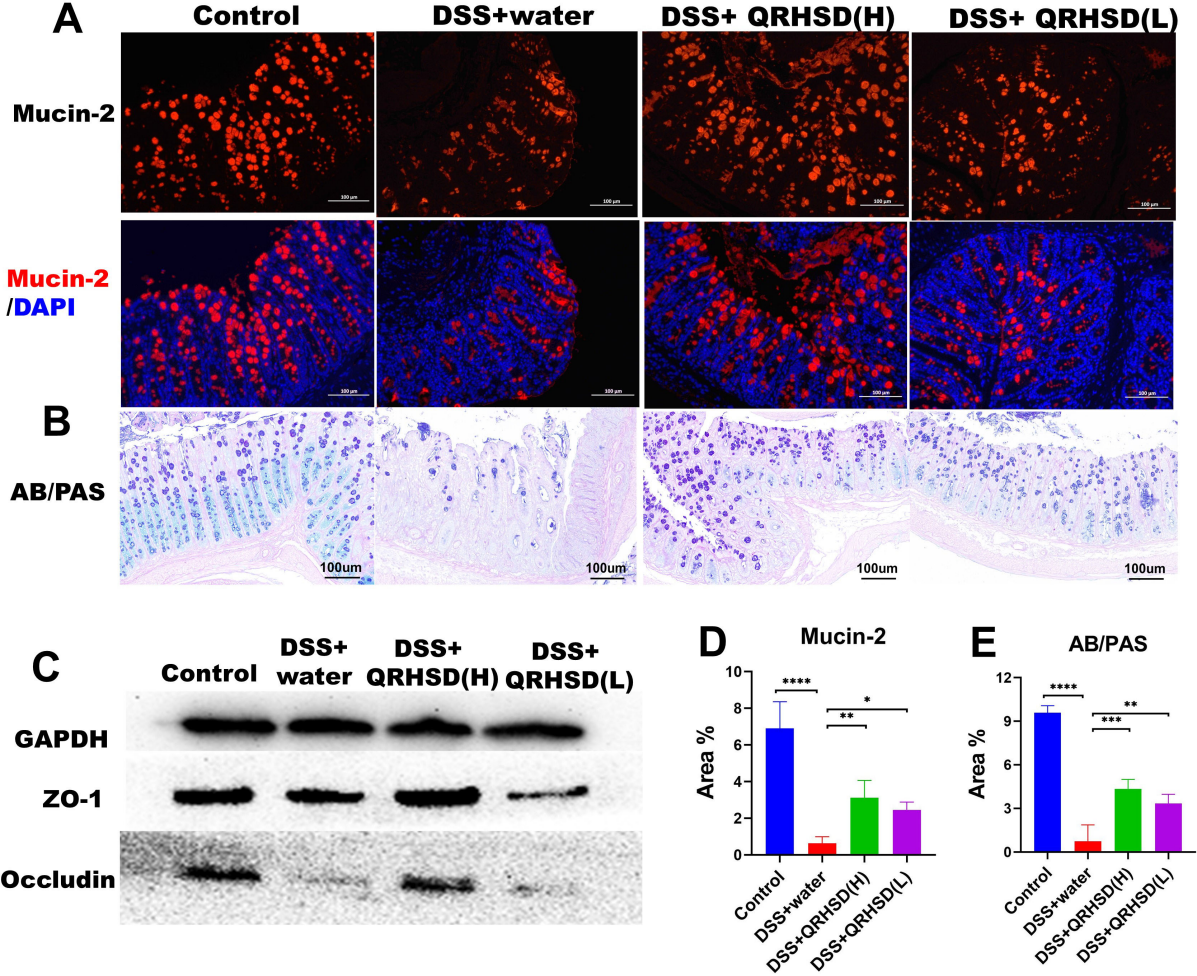
QRHSD strengthened the intestinal barrier in UC mice. Representative pictures and analysis of mucin-2 **(A, D)** and mucin-producing cells **(B, E)**. **(C)** The expressions of occludin and ZO-1 protein were verified by western blot. Data are expressed as means ± SD. n = 7 mice/group, *P < 0.05, **P < 0.01, ***P < 0.001, and ****P <0.0001.

To evaluate the effect of QRHSD on the intestinal barrier integrity, we used immunofluorescence and western blot to detect the expression of intestinal barrier-related proteins in colonic tissues. Immunofluorescence results showed the expression levels of mucin-2 was significantly increased in QRHSD-treated groups (**Figures 4A, D**). Similarly, AB/PAS staining revealed a significant increase in in mucin-2-producing goblet cells in QRHSD-treated groups (**Figures 4B, E**). The expression levels of Occludin and ZO-1 were analyzed by Western blot. DSS treatment significantly reduced Occludin and ZO-1 expression, whereas QRHSD treatment restored their expression levels (**Figure 4C**). These data indicated that QRHSD improved intestinal barrier function in the context of UC.

### 3.5 QRHSD modulated gut microbiota dysbiosis in a UC mouse model

**Figure 5:**
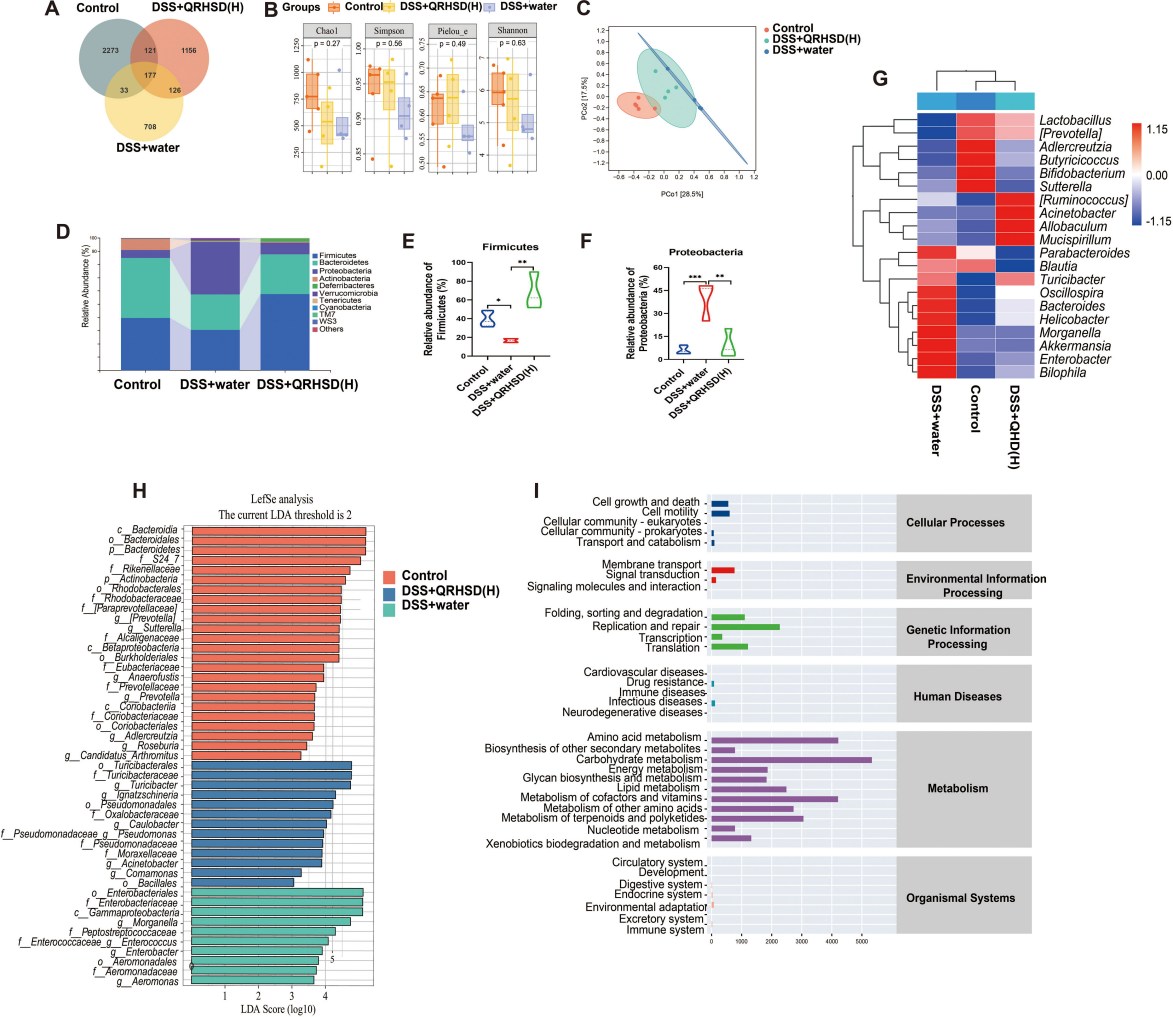
QRHSD modulated gut microbiota dysbiosis in UC mice. **(A)** Venn diagram of OTUs. **(B)** α-diversity analysis based on the Chao, Simpson, Pielou, and Shannon index. **(C)** PCoA plots of β-diversity. **(D)** The relative abundance of predominant bacteria was shown at the phylum level. The relative abundance of Firmicutes **(E)** and Proteobacteria **(F)** among the Control, DSS+water and DSS +QRHSD **(H)** groups. **(G)** The heat maps of gut microbiota at genus level among the Control, DSS+water and DSS +QRHSD **(H)** groups. **(H)** LEfSe comparison of gut microbiota among the Control, DSS+water and DSS +QRHSD **(H)** groups. **(I)** The pathway of different abundances of microflora among the Control, DSS+water and DSS +QRHSD **(H)** groups. n = 4 or 5 mice/group. * *P* < 0.05, **P < 0.01, and *** *P* < 0.001.

To investigate the alterations in the gut microbiota, we examined gut microbiota composition in the Control group, DSS+water group, and DSS +QRHSD (H) group by 16S rRNA gene sequencing. Venn diagram analysis indicated that the three groups shared 177 OTUs, with 2273 OTUs specific to the Control group, 1156 OTUs specific to the DSS+QRHSD (H) group, and 708 OTUs specific to the DSS+water group (**Figure 5A**). The Chao (richness index), Shannon and Simpson (diversity index), and Pielou (evenness index) were used for α-diversity analysis (**Figure 5B**). The Chao, Shannon, Simpson, and Pielou indices were found to be lower in the DSS+water group compared to the Control group; However, all these indices were higher in the DSS+QRHSD(H) group than in the DSS+water group. Nevertheless, no significant difference in α-diversity was observed among the three groups. Principal coordinate analysis (PCoA) was used for β-diversity analysis. As revealed by PCoA (**Figure 5C**), QRHSD treatment significantly affected β-diversity in the gut microbiota, with the microbiota structure in the DSS+QRHSD(H) group differing from that of the DSS+water group. At the phylum level, *Firmicutes* and *Bacteroidetes* were predominant microbial taxa in each group (**Figure 5D**). The relative abundance of *Firmicutes* was lower in the DSS+water group than in the Control group and was elevated by QRHSD (**Figure 5E**). Additionally, the phyla *Proteobacteria* were present in relatively high abundances in the DSS+water group, whereas its abundance was significantly reduced by QRHSD (**Figure 5F**). The relative abundance of top 10 predominant bacteria at the class, order, and family levels was displayed in **Supplementary Figure 1**. The top 20 microflora at genus level among the Control, DSS+water, and DSS+QRHSD(H) groups were shown as a heat map (**Figure 5G**). According to linear discriminant analysis effect size (LEfSe) analysis (LDA > 2), the main representative taxa in the Control, DSS+water, and DSS+QRHSD(H) groups were shown in **Figure 5H**. In addition, the functional prediction of the gut microbiota of the Control, DSS+water, and DSS+QRHSD(H) groups indicated that the major functional pathways of these bacteria were enriched in metabolic processes, including amino acid metabolism, carbohydrate metabolism, metabolism of cofactors and vitamins, and lipid metabolism (**Figure 5I**). Genetic information processing was primarily associated with replication and repair, whereas cellular processes are mainly enriched in cell motility. Taken together, these results demonstrated that modification of gut microbiota may play a crucial role in the protective effects of QRHSD against UC.

### 3.6 QRHSD affected metabolites and metabolic pathways in a UC mouse model

**Figure 6:**
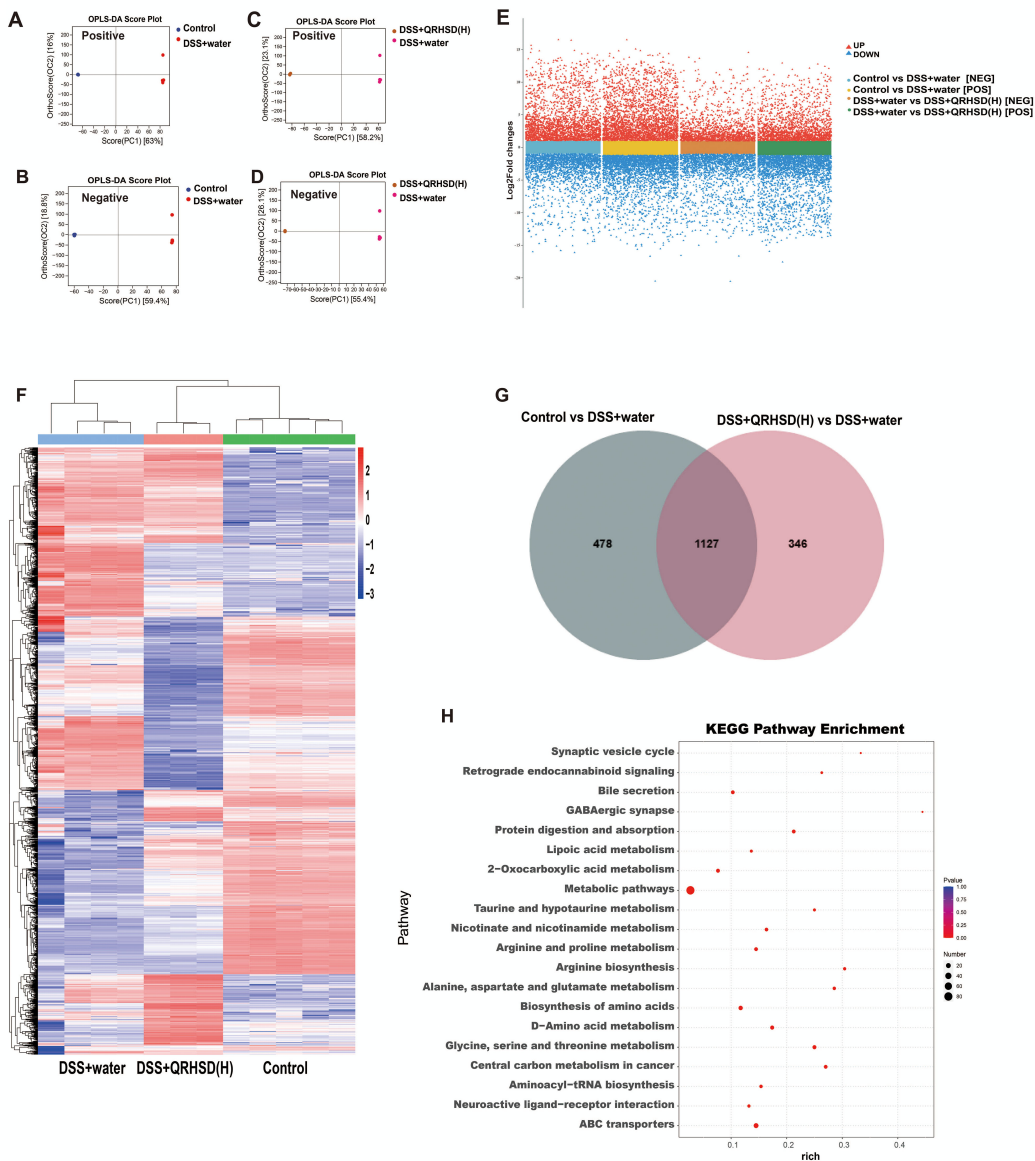
The metabolic profile of mice after QRHSD intervention. **(A, B)** OPLS-DA score plots of positive **(A)** and negative **(B)** ion modes of Control vs DSS+water groups. **(C, D)** OPLS-DA score plots of positive **(C)** and negative **(D)** ion modes of DSS+water vs DSS +QRHSD (H) groups. **(E)** Volcanic plot of differentially expressed metabolites (positive and negative ion modes) of Control vs DSS+water groups, and DSS+water vs DSS +QRHSD **(H)** groups. **(F)** Heat map of identified differential metabolites among the Control, DSS+water and DSS +QRHSD **(H)** groups. **(G)** Venn Diagram of differentially expressed metabolites among the Control, DSS+water and DSS +QRHSD **(H)** groups. **(H)** KEGG analysis of differential metabolites. n = 3-5 mice/group.

Non-targeted metabolomics analysis was performed on the intestinal contents of mice in the Control, DSS+water, and DSS+QRHSD(H) groups. Orthogonal partial leastsquares discriminant (OPLS-DA) analysis was performed separately on metabolites screened in positive and negative ion modes. The results revealed significant differences in the metabolites of the three groups (**Figures 6A–D**). The volcano plot displayed the overall distribution of metabolites in the positive and negative ion modes, with red dots representing significantly up-regulated metabolites and blue dots representing significantly down-regulated metabolites (**Figure 6E**). The differential metabolites among the Control, DSS+water, and DSS+QRHSD(H) groups were shown in a heat map (**Figure 6F**). A total of 1605 differential metabolites were identified between the Control and DSS+water groups, and 1473 differential metabolites were identified between the DSS+water and DSS+QRHSD(H) groups (**Figure 6G**). Among these, 1127 metabolites were common to both comparisons (**Figure 6G**). Of these, 404 metabolites were significantly elevated in the DSS+water group compared to the Control group, and this elevation was reversed by QRHSD treatment. Additionally, 103 metabolites were significantly reduced in the DSS+water group, and this reduction was reversed by QRHSD treatment. KEGG enrichment analyses of the 404 and 103 differential metabolites were shown in **Supplementary Figure 2**, separately. KEGG enrichment analyses of the 507 differential metabolites revealed that metabolic pathways, bile secretion, ABC transporters, and biosynthesis of amino acids were the main enriched pathways (**Figure 6H**).

### 3.7 The molecular action mechanisms of QRHSD on UC were closely related to multiple signaling pathways

**Figure 7:**
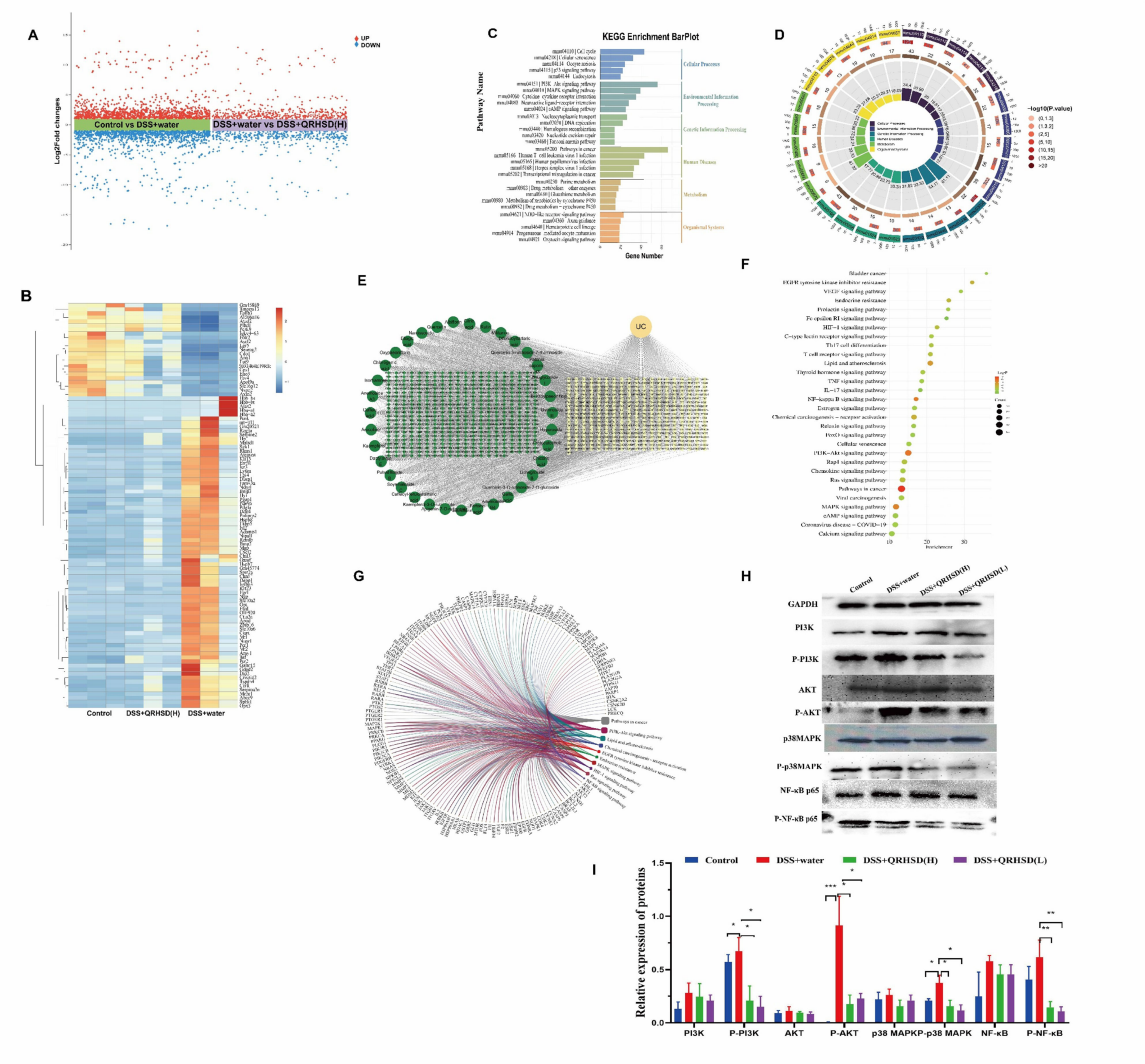
QRHSD treatment of UC is associated with multiple signaling pathways. **(A)** Volcanic plot of the differentially expressed genes of the Control vs DSS+water groups, and DSS+water vs DSS +QRHSD (H) groups. **(B)** Heat map of differential genes among the Control, DSS+water and DSS +QRHSD **(H)** groups. **(C, D)** KEGG analysis of differential genes among the Control, DSS+water and DSS +QRHSD **(H)** groups. **(E)** The interactive network of QRHSD putative targets and known UC targets is shown. **(F)** KEGG pathway analysis of common target genes. **(G)** Chord diagram of major pathways and pathway related genes. **(H)** The protein expressions of PI3K, p-PI3K, AKT, p-AKT, p38MAPK, p-p38MAPK, NF-κB p65, and p-NF-κB p65 in the Control, DSS+water, DSS +QRHSD (H), and DSS +QRHSD (L) groups were analyzed by western blot analysis. **(I)** The statistical results of the Western blot analysis for each protein. n = 3 mice/group. * *P* < 0.05, **P < 0.01, and *** *P* < 0.001.

To better understand the role of QRHSD in UC mice, we sequenced and analyzed the mRNA expression profile of various genes in the Control, DSS + water, and DSS + QRHSD(H) groups. The results revealed that 2247 genes (1112 up-regulated and 1135 down-regulated) were significantly differentially expressed between the Control and DSS+water groups. Additionally, 852 genes (441up-regulated and 411 down-regulated) were significantly differentially expressed between the DSS+water and DSS+QRHSD(H) groups (**Figure 7A**). The top 100 genes that were differentially expressed among the Control, DSS + water, and DSS + QRHSD(H) groups were shown as heatmaps in **Figure 7B**. KEGG pathway analysis was performed to identify the most significantly enriched pathways. The results showed that the most significantly enriched pathways were related to cellular processes, environmental information processing, genetic information processing, human diseases, metabolism, and organismal systems (**Figures 7C, D**). It is worth noting that PI3K/AKT and MAPK signaling pathways are classic pathways associated with inflammation and are involved in the pathogenesis of UC. Moreover, we used network pharmacology to predict the potential targets of UC and QRHSD components. In total, we obtained 362 common targets of QRHSD and UC (**Figure 7E**). KEGG pathway analysis revealed PI3K-AKT and MAPK signaling pathways were the main pathways, which was consistent with the RNA-seq results (**Figure 7F**). Notably, the NF-κB signaling pathway, another classical inflammatory pathway, was also found to be involved in the treatment of UC by QRHSD. Genes associated with these three pathways were predicted and are displayed in **Figure 7G**. Western blot was used to detect pathway-associated proteins, and the results were exhibited in **Figures 7H, I**.

**Figure 8:**
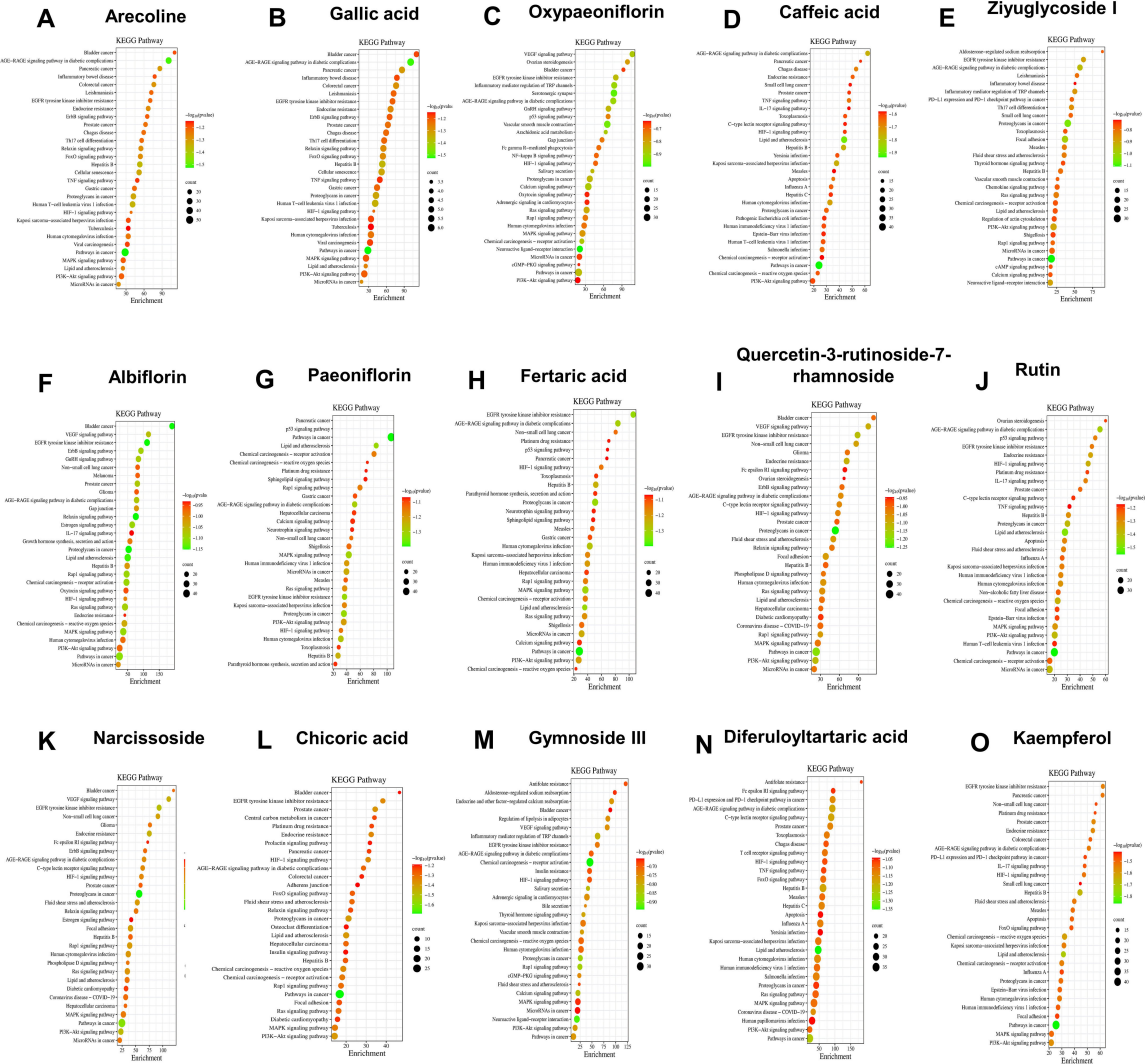
KEGG pathway analysis of common target genes in UC and QRHSD components, including arecoline **(A)**, gallic acid **(B)**, oxypaeoniflorin **(C)**, caffeic acid **(D)**, ziyuglycoside I **(E)**, albiflorin **(F)**, paeoniflorin **(G)**, fertaric acid **(H)**, quercetin-3-rutinoside-7-rhamnoside **(I)**, rutin **(J)**, narcissoside **(K)**, chicoric acid **(L)**, gymnoside III **(M)**, diferuloyltartaric acid **(N)**, and kaempferol **(O)**.

Further, we also used network pharmacology to predict the components that might play a major role in QRHSD. As shown in **Figure 8**; **Supplementary Figure 3**, we obtain the possible pathways of each component in QRHSD acting on UC. Among them, the compounds arecoline, gallic acid, oxypaeoniflorin, caffeic acid, ziyuglycoside I, albiflorin, paeoniflorin, fertaric acid, quercetin-3-rutinoside-7-rhamnoside, rutin, narcissoside, chicoric acid, gymnoside III, diferuloyltartaric acid, and kaempferol may be the main components of QRHSD for the treatment of UC, and it was predicted that these compounds exerted their therapeutic effects on UC through inflammatory signaling pathways such as PI3K/AKT, MAPK, NF-κB, which is consistent with the pathway of QRHSD in treating UC. These results suggested that QRHSD treated UC by acting on multiple signaling pathways, which also validates the “multi-pathways” and “multi-targets” characteristics of herbal medicine.

### 3.8 Correlation analysis among gut microbiota, metabolites, and UC-related factors

**Figure 9:**
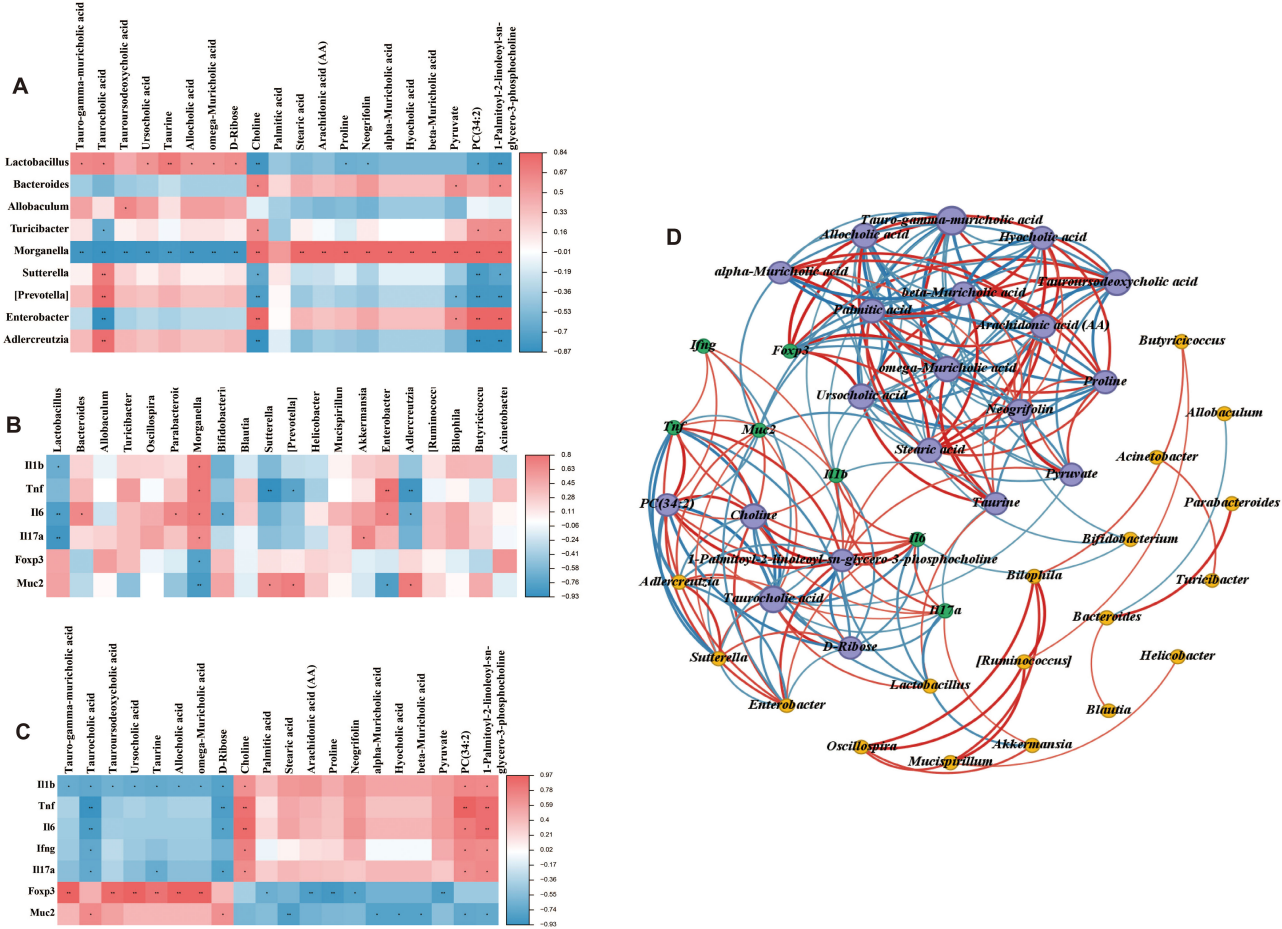
Correlation analysis among gut microbiota, metabolites and UC-related factors. **(A)** Correlation between the top 20 intestinal contents metabolites and gut microbiota at the genus level. **(B)** Correlation between UC-related clinical factors and gut microbiota at the genus level. **(C)** Correlation between UC-related clinical factors and the top 20 intestinal contents metabolites. **(D)** Correlation among UC-related clinical factors, the top 20 intestinal contents metabolites, and gut microbiota at the genus level. The red line indicates a positive correlation, the blue line indicates a negative correlation, and the thickness of the line indicates the strength of the correlation. *P < 0.05, and **P < 0.01.

Spearman’s correlation analysis was employed to generate correlation heatmaps, enabling the exploration of relationships among the top 20 intestinal content metabolites, genus flora, and UC clinical correlates (IL-1β, TNF-α, IL-6, IFN-γ, IL-17A, foxp3, and mucin-2). As depicted in **Figure 9A**, at the genus level, tauro-gamma-muricholic acid, taurocholic acid, ursocholic acid, taurine, allocholic acid, omega-muricholic acid, and D-ribose exhibited positive correlations with *Lactobacillus*, which was significantly reduced in UC mice. In contrast, metabolites choline, proline, neogrifolin, PC(34:2), and 1-Palmitoyl-2-linoleoyl-sn-glycero-3-phosphocholine showed negative correlations with *Lactobacillus*. Conversely, tauro-gamma-muricholic acid, taurocholic acid, tauroursodeoxycholic acid, ursocholic acid, taurine, allocholic acid, omega-muricholic acid, and D-ribose were negatively correlated with *Morganella*, which was significantly elevated in UC mice. Meanwhile, stearic acid, arachidonic acid (AA), proline, neogrifolin, alpha-muricholic acid, hyocholic acid, beta-muricholic acid, pyruvate, PC (34:2), and 1-Palmitoyl-2-linoleoyl-sn-glycero-3-phosphocholine were positively correlated with *Morganella*. The relationship between the gut microbiota and clinical factors was illustrated in **Figure 9B**. It was observed that the gut microbiota significantly influenced the expression of clinical factors. Specifically, *Lactobacillus* was negatively correlated with UC-related proinflammatory factors, whereas *Morganella* exhibited positive correlations with most UC-related proinflammatory factors, and negative correlations with Foxp3 and mucin-2. Similarly, intestinal content metabolites also showed strong correlations with UC-related factors (**Figure 9C**). For instance, metabolites that were reduced in UC mice were negatively correlated with IL-6. Furthermore, Spearman’s algorithm (threshold setting: IRI > 0.6, P < 0.05) was applied to analyze the triple correlation among the top 20 gut content metabolites, genus-level microbiota, and UC-related clinical factors. As shown in **Figure 9D**, the node size represents the number connections associated with each node, with larger nodes indicating more connections. The red lines denote positive correlations, blue lines indicate negative correlations, and the line thickness reflects the strength of the correlation. These findings underscored the intricate interplay between gut content metabolites, gut microbiota, and UC-related clinical factors.

## 4 Discussion

**Figure 10:**
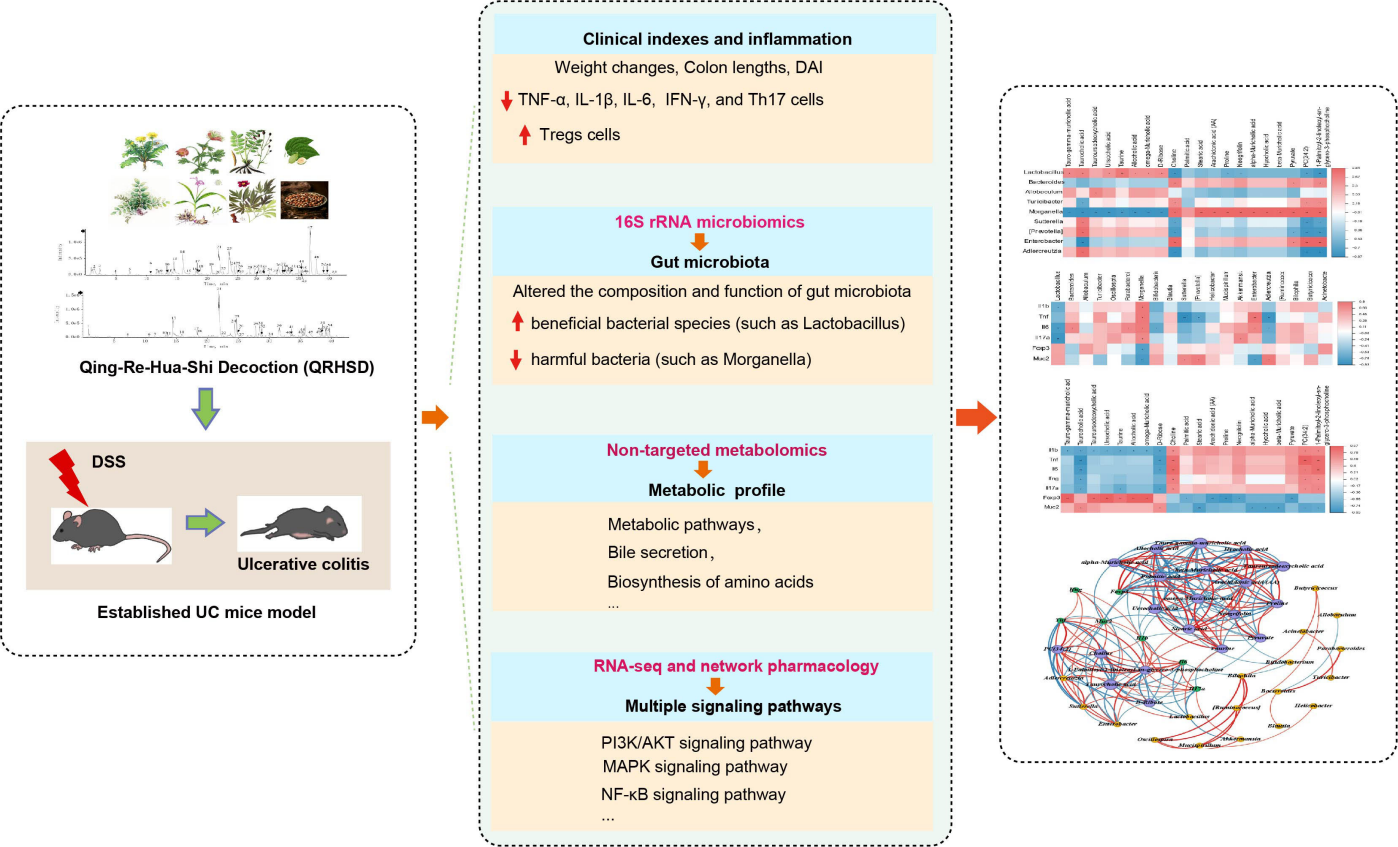
Multi-omics analysis revealed the protective effect of QRHSD on DSS-induced UC.

In this study, we systematically investigated the therapeutic effects of QRHSD on DSS-induced colitis in mice. Our findings revealed that QRHSD treatment effectively attenuated clinical disease activity and reduced excessive inflammation. Importantly, it demonstrated significant mucosal repair capabilities and restored the Th17/Treg balance in the colitis model. Through comprehensive multi-omics analysis, we identified that QRHSD treatment induced substantial alterations in both gut microbial composition and metabolite profiles, while simultaneously regulating multiple signaling pathways. Furthermore, our study revealed significant correlations among gut microbiota composition, metabolite profiles, and UC-related clinical parameters, which may be attributed to the therapeutic effects of QRHSD intervention (as shown in **Figure 10**). These findings provide a theoretical foundation for the application of QRHSD in UC treatment. However, the precise mechanisms underlying these complex interactions require further investigation through subsequent studies to fully elucidate the intricate relationships between gut microbial communities, metabolic alterations, and clinical manifestations in UC pathogenesis and treatment.

Treg and Th17 cells, which are differentiated from naïve CD4^+^T cells, play critical roles in the pathogenesis of UC (Chang et al., 2021). Clinical studies have consistently demonstrated a marked elevation in the population of Th17 cells within the inflamed intestinal mucosa of patients with inflammatory bowel disease (IBD) compared to non-inflamed tissue (Fujino et al., 2003; Sugihara et al., 2010; Jiang et al., 2014; D’Ambrosio et al., 2016). Conversely, a reduction in the population of regulatory T cells (Tregs) has been observed in IBD patients, as evidenced by multiple studies (Maul et al., 2005; Pedros et al., 2016). Supporting these clinical findings, experimental studies in animal models have revealed that the depletion of Tregs in intestinal mucosa exacerbated DSS-induced colitis, whereas the transfer of Tregs into colitis mice alleviated colitis (Mottet et al., 2003; Zhang et al., 2016). In the present study, we demonstrated that QRHSD treatment significantly increased the proportion of CD4^+^CD25^+^Foxp3^+^ cells and decreased the percentage of Th17 cells in the colonic tissue of UC mice. These data suggested that QRHSD exerts its therapeutic effects by modulating the balance between Treg and Th17 cell populations, thereby attenuating inflammation and restoring immune homeostasis in the colonic microenvironment of UC mice.

Previous studies have underscored the pivotal role of gut microbiota in the pathogenesis of UC (Guo et al., 2020; Wang et al., 2022). Evidence derived from both UC patients and animal models has demonstrated that dysbiosis of gut microbiota plays a critical role in the initiation and perpetuation of colonic inflammation (Lepage et al., 2011; Samanta et al., 2012; Khan et al., 2019). Interventions such as prebiotics, probiotics, and synthetic probiotics have been shown to confer therapeutic benefits to specific subgroups of IBD patients (Damaskos and Kolios, 2008). Investigations in IBD, including those with UC, have revealed a marked reduction in the abundance of *Firmicutes* and a concomitant increase in *Proteobacteria* (Macpherson et al., 2000; Frank et al., 2007; Sartor, 2008), alongside a significant decline in beneficial bacterial species from the genera *Bacteroidetes* and *Lactobacillus* (Nemoto et al., 2012; Sha et al., 2013). In alignment with these findings, our study observed that UC mice exhibited a decreased microbial diversity of *Firmicutes* and an increased diversity of *Proteobacteria* at the phylum level, which was reversed by QRHSD treatment. Furthermore, QRHSD administration significantly elevated the abundance of beneficial bacterial species such as *Lactobacillus*, and decreased the abundance of potentially pathogenic genera like *Morganella* at genus levels. Additionally, QRHSD treatment modulated the overall diversity of the intestinal community in UC mice. These results collectively suggest that the therapeutic efficacy of QRHSD in UC is closely linked to its ability to restore gut microbiota homeostasis.

In recent years, the interplay between gut microbiota and metabolites has emerged as a pivotal area of research in elucidating the mechanisms underlying anti-UC therapies (Yang and Cong, 2021). In this study, we screened for metabolites by performing untargeted metabolomic assays on contents from the gut. The top 20 metabolites mainly consisted of Tauro-gamma-muricholic acid, Taurocholic acid, Tauroursodeoxycholic acid, Ursocholic acid, Taurine, Allocholic acid, omega-Muricholic acid, D-Ribose, Choline, Palmitic acid, Stearic acid, Arachidonic acid (AA), Proline, Neogrifolin, alpha-Muricholic acid, Hyocholic acid, beta-Muricholic acid, Pyruvate, PC(34:2), and 1-Palmitoyl-2-linoleoyl-sn-glycero-3-phosphocholine. These metabolites were primarily associated with pathways such as metabolic pathways, bile secretion, and taurine and hypotaurine metabolism, et al. Among them, AA, an n-6 polyunsaturated fatty acid (n-6 PUFA), has been implicated in UC pathogenesis. Studies have reported a significantly higher risk of UC in individuals with elevated levels of AA in adipose tissue (de Silva et al., 2010). Taurine (Tau), a semiessential amino acid in mammals, has demonstrated preventive and therapeutic effects in various intestinal disorders. A randomized controlled trial revealed that UC patients in remission who adhered to an anti-inflammatory diet exhibited higher fecal taurine levels compared to controls (Keshteli et al., 2022). Furthermore, taurine deficiency has been shown to increase the susceptibility to DSS-induced colitis, while taurine pretreatment protected against experimental colitis in mice by enhancing intestinal barrier function and suppressing the TLR4/NF-κB pathway (Zheng et al., 2024). Bile salts, which play an important role in regulating epithelial cell viability in the gastrointestinal lumen (Stenman et al., 2013), are dysregulated in IBD patients, correlating with disturbances in bile salt metabolism (Yang and Cong, 2021). Tauroursodeoxycholic acid (TUDCA), the taurine-coupled conjugate of ursodeoxycholic acid, has been shown to inhibit experimental colitis by preventing early intestinal epithelial cell death (Laukens et al., 2014). Recent studies further demonstrated that TUDCA liposome alleviated DSS-induced ulcerative colitis, restored the intestinal barrier, and promoted the ecological restoration of the gut microbiota (Zhao et al., 2024). Consistent with these findings, DSS-induced UC mice exhibited significantly elevated levels of β-muricholic acid (βMCA) and markedly reduced levels of TUDCA (Liu et al., 2022b), aligning with our observations. In this study, we also focused on the correlation between the gut microbiota and significantly altered metabolites in UC mice treated with QRHSD. *Lactobacillus* showed a positive correlation with bile acid metabolites (Tauro-gamma-muricholic acid, Taurocholic acid, Taurine, Allocholic acid, omega-Muricholic acid), suggesting that the increase in *Lactobacillus* may be related to bile acid metabolism. In contrast, *Morganella* exhibited strong negative correlations with several metabolites, particularly bile acids, indicating that an increase in *Morganella* might be linked to reduced levels of these metabolites, potentially exerting adverse effects on the gut environment. *Bacteroides* and *Turicibacter* showed weak correlations with most metabolites, suggesting that they may have minimal influence on these specific metabolites. Additionally, *Enterobacter* and *[Prevotella]* also displayed weak correlations, implying that they may play a limited role in the metabolism of these metabolites. However, these findings are preliminary, and the intricate relationships between gut microbiota, individual bacterial species, and their associated metabolites necessitate further comprehensive investigation and validation. Collectively, our findings implied that the protective effect of QRHSD in alleviating colitis was closely linked to its modulation of gut microbiota and their metabolites.

Accumulating studies have demonstrated that modulation of key molecular signaling pathways, including PI3K/AKT, NF-κB, and MAPK, plays a critical role in controlling the development of intestinal inflammation in UC and restoring intestinal homeostasis. Among these pathways, NF-κB, a multifunctional nuclear transcription factor, regulated a diverse spectrum of biological processes, including immune and inflammatory regulation (Lu and Zhao, 2020). Extensive studies have established that NF-κB activation mediated intestinal inflammatory responses during the pathogenesis of UC, positioning the NF-κB signaling pathway as a key therapeutic target for UC treatment (Zheng et al., 2022). For example, the Xianglian pill, a traditional Chinese patent medicine, has been shown to alleviate UC by modulating the TLR4/MyD88/NF-κB signaling pathway (Dai et al., 2023). Similarly, Gegen Qinlian decoction suppressed the hyperactive immune response by inhibiting TLR4/NF-κB signaling in acute colitis mice (Li et al., 2016), and regenerated the colonic mucosa via bidirectional regulation of Notch signaling in acute/chronic UC models (Zhao et al., 2020). Additionally, Shaoyao decoction has demonstrated protective effects against UC by mitigating pyroptosis via inhibition of the MKP1/NF-κB/NLRP3 pathway (Wei et al., 2021). The PI3K/AKT pathway, another critical signaling cascade, is integral to numerous cellular processes, including cell proliferation, autophagy, survival, and differentiation (Ma et al., 2023). Activation of the PI3K/AKT pathway has been implicated in UC pathogenesis, and its inhibition by herbal formulations has shown therapeutic potential. For example, Kuijieyuan Decoction has been found to improved ameliorate intestinal barrier injury in UC by affecting TLR4-dependent PI3K/AKT/NF-κB signaling (Liu et al., 2020). Renshen Baidu powder has been shown to inhibit the phosphorylation of PI3K, AKT, and NF-κB, while upregulating the NF-κB inhibitor IκB and downregulating the activation protein IKK, thereby blocking the PI3K/AKT/NF-κB signaling pathway and alleviating UC symptoms (Ye et al., 2022). The mitogen-activated protein kinase (MAPK) signaling pathway, which regulates cell growth, migration, metabolism, differentiation, and apoptosis, has also been implicated in UC pathogenesis (Liu et al., 2022a). Activation of the p38 MAPK pathway has been linked to IBD (Long et al., 2022), and Our previous research demonstrated that p38α deficiency in macrophages ameliorated murine experimental colitis by modulating inflammatory and immune process (Chen et al., 2022). Traditional herbal medicines, characterized by their multi-component and multi-target nature, offer a unique therapeutic approach for complex diseases like UC. In this study, we observed that QRHSD exerted a significant regulatory effect on the PI3K-AKT/MAPK/NF-κB signaling pathway in DSS-induced colitis mice. These findings provide mechanistic evidence supporting the multi-targeted therapeutic potential of QRHSD in the treatment of UC.

QRHSD represents a promising therapeutic approach for ulcerative colitis (UC), offering a unique multi-targeted mechanism that distinguishes it from conventional treatments. Unlike standard UC therapies, QRHSD, as a traditional Chinese herbal formulation, addresses UC through a holistic approach, targeting not only inflammation but also intestinal barrier integrity, gut microbiota balance, and metabolic regulation. In contrast to antibiotics or probiotics, which are sometimes used as adjunctive therapies in UC treatment, QRHSD appears to restore microbial homeostasis through a more nuanced mechanism, potentially promoting the growth of beneficial bacteria while suppressing pathogenic species. Furthermore, QRHSD demonstrates significant anti-inflammatory properties by targeting multiple signaling pathways, such as PI3K/AKT, MAPK, and NF-κB, which are central to the pathogenesis of UC. Additionally, as a natural formulation, QRHSD is likely to exhibit a more favorable safety profile compared to conventional UC medications, making it a viable option for long-term use or as an adjunct to standard therapies. Nonetheless, it must be acknowledged that the mechanisms and therapeutic efficacy of QRHSD are still under investigation. Further research is needed to elucidate the mechanisms by which QRHSD acts on UC, and large-scale clinical trials should be conducted to compare its efficacy with existing treatments for UC.

## 5 Conclusion

In conclusion, this study demonstrated that QRHSD effectively alleviated DSS-induced colitis in mice. The beneficial effects of QRHSD on intestinal mucosal barrier disruption and colonic inflammation were at least partially mediated through modulation of intestinal microbiota imbalance, intestinal metabolites, and multiple signaling pathways. These findings elucidate a novel mechanistic basis for the therapeutic efficacy of traditional Chinese medicine in the treatment of UC, highlighting its potential as a multi-targeted intervention strategy.

## Data Availability

The datasets presented in this study can be found in online repositories. The names of the repository/repositories and accession number(s) can be found below: https://www.ncbi.nlm.nih.gov/, PRJNA1223290 https://www.ncbi.nlm.nih.gov/, PRJNA1234055.
