# Supplementary material for: Qing-Re-Hua-Shi Decoction ameliorates DSS-induced colitis by modulating multiple signaling pathways and remodeling the gut microbiota and metabolite profile
Source: Front Cell Infect Microbiol. 2025 Apr 2;15:1541289. doi: 10.3389/fcimb.2025.1541289 (PMC11999956; doi:10.3389/fcimb.2025.1541289)
Supplement: Supplementary file 1 [file DataSheet1.pdf]

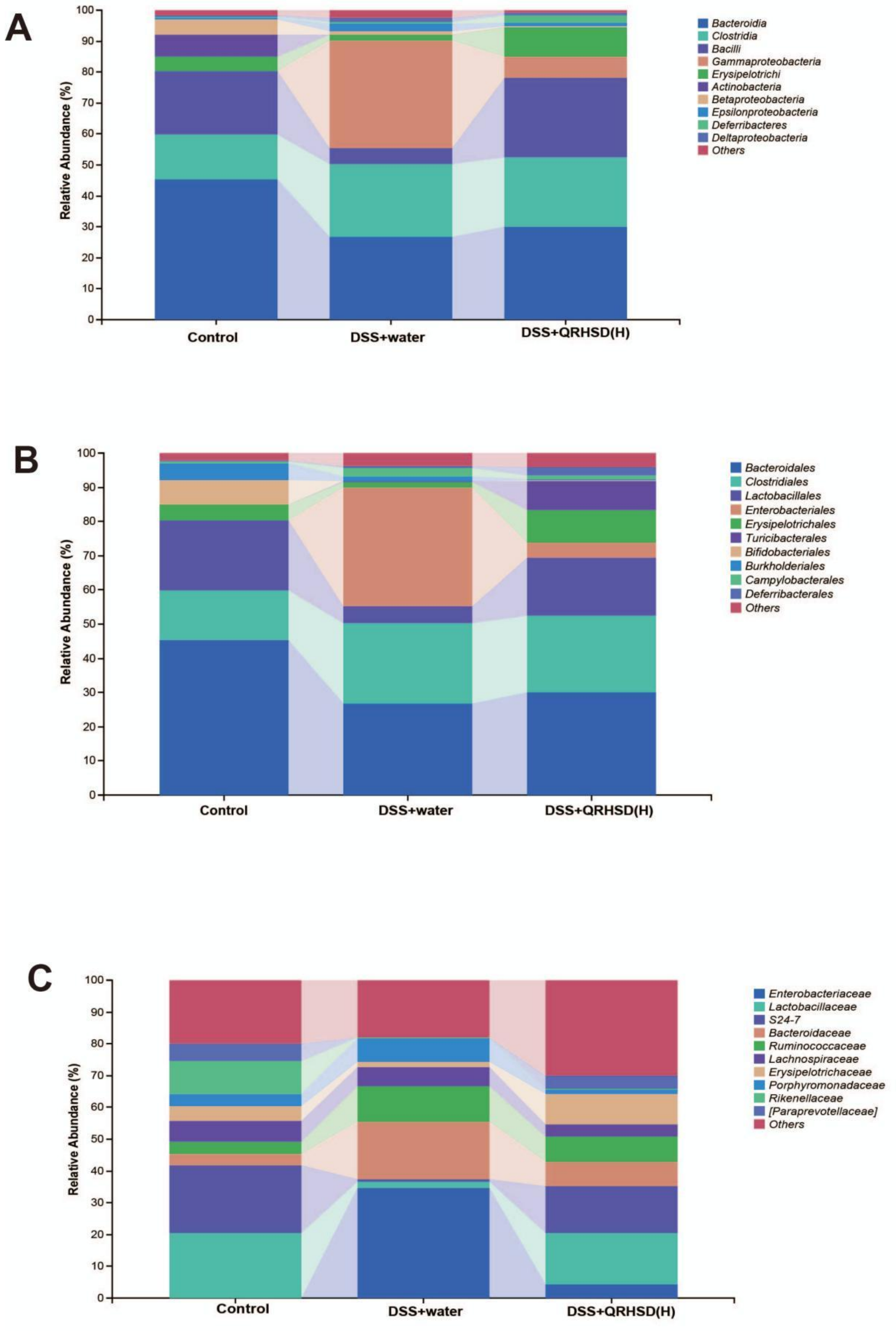

Supplementary Fig. 1 The relative abundance of top 10 predominant bacteria at the at the class **(A)**, order **(B)**, and family **(C)** levels was displayed separately.

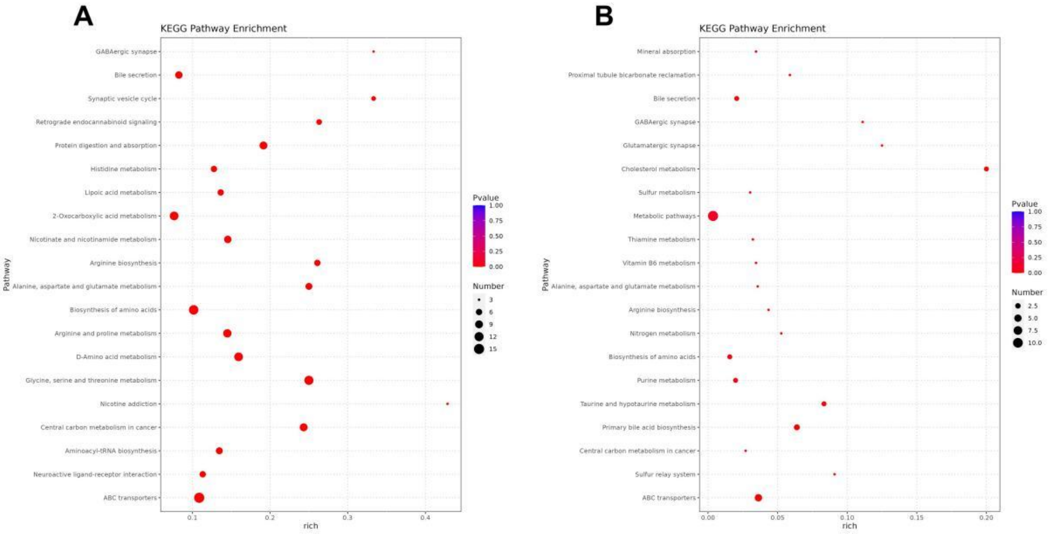

Supplementary Fig. 2 (A) KEGG enrichment analyses of the 404 differential metabolites. **(B)** KEGG enrichment analyses of the 103 differential metabolites.

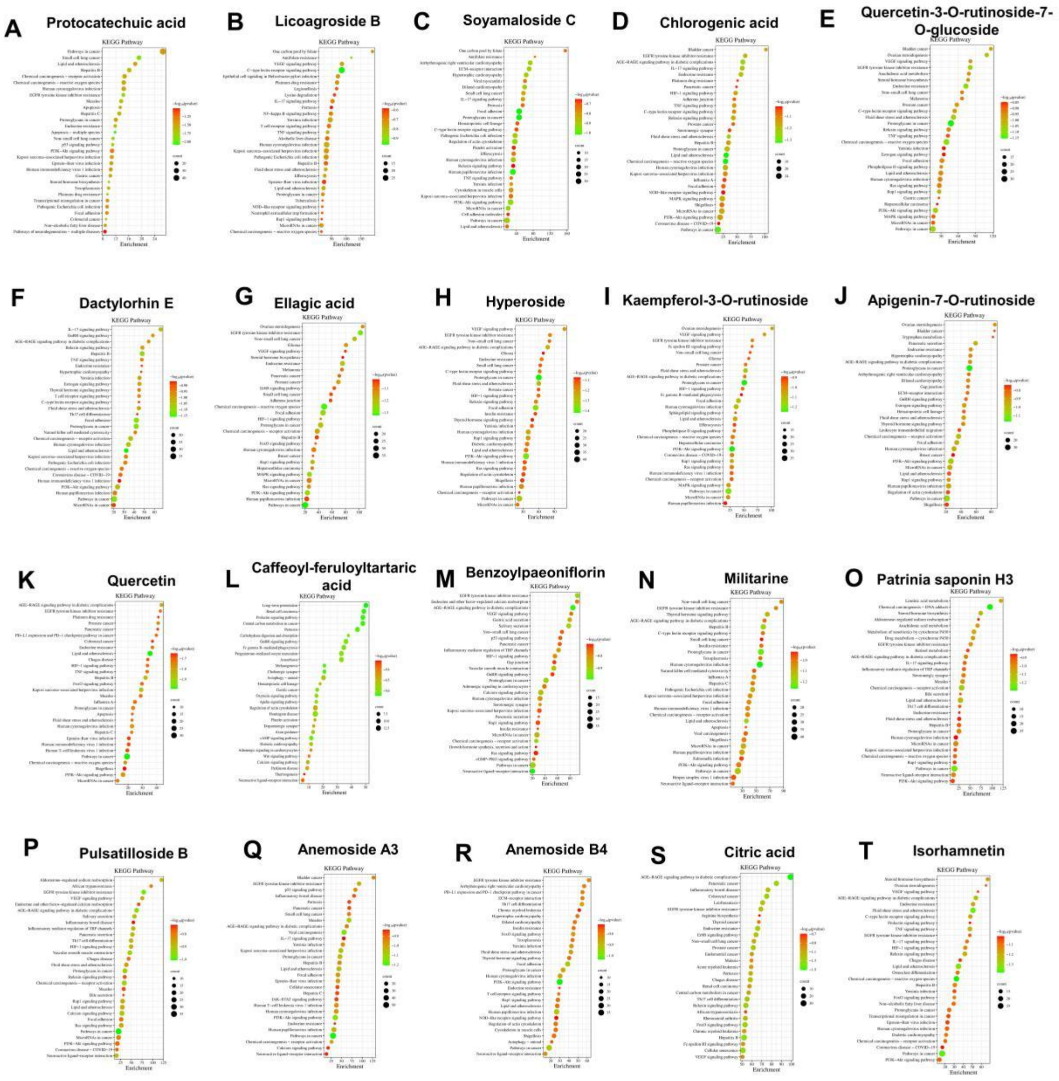

Supplementary Fig. 3 KEGG pathway analysis of common target genes in UC and QRHSD components, including protocatechuic acid **(A)**, licoagroside B **(B)**, soyamaloside C **(C)**, chlorogenic acid **(D)**, quercetin-3-O-rutinoside-7-O-glucoside **(E)**, dactylorhin E **(F)**, ellagic acid **(G)**, hyperoside **(H)**, kaempferol-3-O-rutinoside **(I)**, apigenin-7-O-rutinoside **(J)**, quercetin **(K)**, caffeoyl-feruloyltartaric acid **(L)**, benzoylpaeoniflorin **(M)**, militarine **(N)**, patrinia saponin H3 **(O)**, Pulsatilloside B **(P)**, Anemoside A3 **(Q)**, Anemoside B4 **(R)**, Citric acid **(S)**, and Isorhamnetin **(T)**.

Supplementary table 1 Mobile phase gradient

Time (min)	A%	B%
0～3	3	97
3～7	3～6	97～94
7～15	6～13	94～87
15～30	13～25	87～75
30～35	25～40	75～60
35～43	40～60	60～40
43～48	60～90	40～10
48～51	90	10
51～51.1	90～3	10～97
51.1～54	3	97
